# Metabolic effects of agro-infiltration on *N. benthamiana* accessions

**DOI:** 10.1007/s11248-021-00256-9

**Published:** 2021-04-28

**Authors:** Margit Drapal, Eugenia M. A. Enfissi, Paul D. Fraser

**Affiliations:** Biochemistry, Royal Holloway University of London, Egham, UK

**Keywords:** Agroinfiltration, *N. benthamiana*, Metabolite analysis, Pathogen-defence

## Abstract

Over the recent years, *Nicotiana benthamiana* has gained great importance as a chassis for the production of high value, low volume pharmaceuticals and/or active pharmaceutical ingredients (APIs). The process involving infiltration of the *N. benthamiana* leaves with *Agrobacterium* spp, harbouring vectors with the gene of interest, facilitates transient expression. To date, little information is available on the effect of the agro-infiltration process on the metabolome of *N. benthamiana,* which is necessary to improve the process for large-scale, renewable manufacturing of high value compounds and medical products. Hence, the objective of the present study was to assess metabolic adaptation of *N. benthamiana* as a response to the presence of *Agrobacterium*. The present study elucidated changes of the steady-state metabolism in the agroinfiltrated leaf area, the area around the infection and the rest of the plant. Furthermore, the study discusses the phenotypic advantages of the *N. benthamiana* lab strain, optimised for agro-infiltration, compared to three other wild accessions. Results showed that the lab strain has a different metabolic composition and showed less alterations of the phenylpropanoid pathway and cell wall remodelling in the agroinfiltrated leaf areas, for example chlorogenic acid, cadaverine and C18:0–2-glycerol ester. In conclusion, both of these alterations present potential candidates to improve the phenotype of the *N. benthamiana* lab strain for a more efficient transient expression process.

**Supplementary Information:**

The online version contains supplementary material available at 10.1007/s11248-021-00256-9.

## Introduction

In recent years, several therapeutics produced in plants have been successfully commercialised. These products include the influenza (H1N1 and H5N1) vaccines and ZMapp, an anti-Ebola antibody cocktail (Landry et al. 2014; Chen and Davis 2016). One of the advantages of plant based production is the rapidity from proof of concept to scalable manufacture. Potentially, these approaches could also be directly transferable to our present attempts to combat the COVID-19 pandemic, through the manufacture of vaccines and therapeutic antibodies (Shanmugaraj et al. 2020; Rosales-Mendoza 2020). The plant chassis of choice for the production of these medical products is *Nicotiana benthamiana* (Goodin et al. 2008; Bally et al. 2018). This *Nicotiana* species is amenable to agroinfiltration and high level transient gene expression. In addition to pharmaceutical proteins, *N. benthamiana* has also been exploited as a cell factory for high value or speciality chemicals (Sathish et al. 2018). Fundamentally, it is also an invaluable resource for determining subcellular localisation and protein interactions. In terms of production in source tissues, the *Agrobacterium* mediated transient expression in *N. benthamiana* has become the cell factory of choice and the *N. benthamiana* laboratory strains LAB and RA4 have become a feature of most biotechnological laboratories and companies working in the field (Krenek et al. 2015; Bond et al. 2016). Ecologically, *N. benthamiana* is native to Australia and has been discovered in different geographical habitants (zones) of the continent. Five accessions are named with respect to their regional origins: Northern Territory (NT), North Western Australia (NWA), Western Australia (WA), Queensland (QLD) and South Australia (SA). The Laboratory (LAB or RA4) evolved strains are closest to the SA ecotype (Bally et al. 2015). It has been shown that the LAB strain contains a mutation in the RNA-dependent RNA polymerase gene, *Rdr1* when compared to wild accessions; this means the *Rdr1* gene product is non-functional and more susceptible to viruses; while the QLD, NWA and WA possess a functioning *Rdr1* conferring greater tolerance (Wylie et al. 2015; Bally et al. 2018).

Despite the biotechnological utility of *N. benthamiana* across multiple industrial sectors the “omic” resources available have been poor. It is only recently that good quality annotated genomes have been generated (Kourelis et al., 2019; https://nbenth.com). Other international programmes are underway to generate new molecular pharming chassis with altered glycosylation profiles and reduced protease activity (Newcotiana project: https://newcotiana.org/). To date, very few comprehensive metabolomic studies have been carried out with *Nicotiana*. Despite the fact that upon agro-infection, metabolism will be affected and metabolic reprogramming could occur in some cases.

The objectives of the present study were to described the *N. benthamiana* metabolome and the effects of *Agrobacterium* infiltration on the steady-state metabolism. The latter objective included a comparison of the lab strain to the wild accessions to elucidate whether (i) the metabolic response to *Agrobacterium* is common to *N. benthamiana* and (ii) whether the common response varies in intensity between the *N. benthamiana* accessions. The present data are discussed in terms of engineering future *N. benthamiana* chassis for optimal renewable production and improved downstream processing of therapeutic proteins and high-value small molecules. The present study highlighted metabolic changes of primary metabolism (TCA cycle, sugars and GABA) and precursors for cell wall remodelling (fatty acids, phenolics and chlorogenic acids) in response to the agroinfiltration. These changes have been detected in all four *N. benthamiana* accessions. However, the lab strain showed a significantly reduced response to the agroinfiltration compared to the wild accessions.

## Experimental procedures

### Plants and bacteria

*Nicotiana benthamiana* Queensland (QLD), Northwest Australia (NWA), West Australia (WA) and lab isolate RA4 (Purchased from Herbalistics Pty Ltd, Australia). Plants were potted with Levington® Advance Pot&Bedding Compost M3 (ICL Specialty Fertilizers, UK) and grown in the glasshouse at 24 °C under supplementary lightning (16 h light/8 h dark cycle) for 6 weeks. The *Agrobacterium* strain LBA4404 with plasmid pAL4404 was cultivated and prepared for agroinfiltration as previously described (Bird et al. 1988). The overnight culture of agrobacteria was centrifuged and the pellet resuspended in infiltration media (10 mM MgCl2, 10 mM MES stock, 200 μM acetosyringone; pH 5.6) to an OD600 of 0.5.

### Agroinfiltration

Sample sets consisted of twelve biological replicates for each accession, except for *N. benthamiana* WA which had eight replicates. Half of the plants were used for control purposes without treatment. For the other half, three leaves per replicate were infiltrated with agrobacterium (~ 0.5-1 mL) to fill approximately half the leaf. This needed two infiltration positions for most of the leaves and was traced on the bottom of the leaves. After four days, leaves were harvested and the traced infiltrated area was separated from the rest of the leaf and kept separately. Triplicates of the four conditions – infiltrated area (I), area around infiltrate (A), non-infiltrated leaf (N) and control leaf (C) – were pooled for each biological replicate. Leaves were frozen in liquid nitrogen immediately after harvest, lyophilised and ground to a fine powder.

### Metabolite extraction and analysis

A portion of the samples (10-11 mg) was weighed out and extracted with methanol/water (800 µL; 1:1) for one hour before phase separation with chloroform (800µL) and centrifugation at full speed for 5 min. An aliquot of the polar extract was prepared with internal standard genistein (1 µg) for LC–MS analysis as previously described (Drapal et al. 2020). LC–MS analysis was based on the protocol published by Drapal et al. (2020) with the following changes: flow of 0.3 ml/min, injection volume (1 µL) and the sample was transferred directly from the C18 column to the MS system. The solvent gradient was adapted to start at 95% (A) for 1 min, followed by a linear decrease to 70% (A) at 6 min, 0% (A) at 7.5 min, which was held until 9 min before return to initial conditions of 95% (A) at 10.5 min. After the last step, the column was re-equilibrated for 1.5 min.

Aliquots of the polar and non-polar phase (150 and 700 µL, respectively) were dried down with _d4_-suiccinic acid (10 µg) or _d27_-myristic acid (10 µg), respectively; derivatised and analysed by GC–MS in split-less mode as previously described (Drapal et al. 2019).

### Data analysis

GC–MS data was processed with AMDIS (V2.71) and LC–MS data with Agilent Profinder (V10.0 SP1, Agilent Technologies, Inc.). Metabolites for both data sets were identified with an in-house library (Supplementary Table 4)and comparison of unknown peaks to previous publications (Bhattacharya et al. 2010; Ncube et al. 2014) or database NIST11 (http://chemdata.nist.gov/mass-spc/ms-search/). The metabolite data was relatively quantified to the respective internal standard and sample weight. Statistical analysis was performed with Simca P (13.0.3.0, Umetrics), XLSTAT (2017, Addinsoft), and Metaboanalyst (Xia and Wishart 2016). Data was subjected to auto-scaling for analysis with Metaboanalyst. Non-parametric ANOVA was performed due to the small sample size and included false discovery rate correction for multiple comparisons.

## Results

### *N. benthamiana* metabolome

**Fig. 1:**
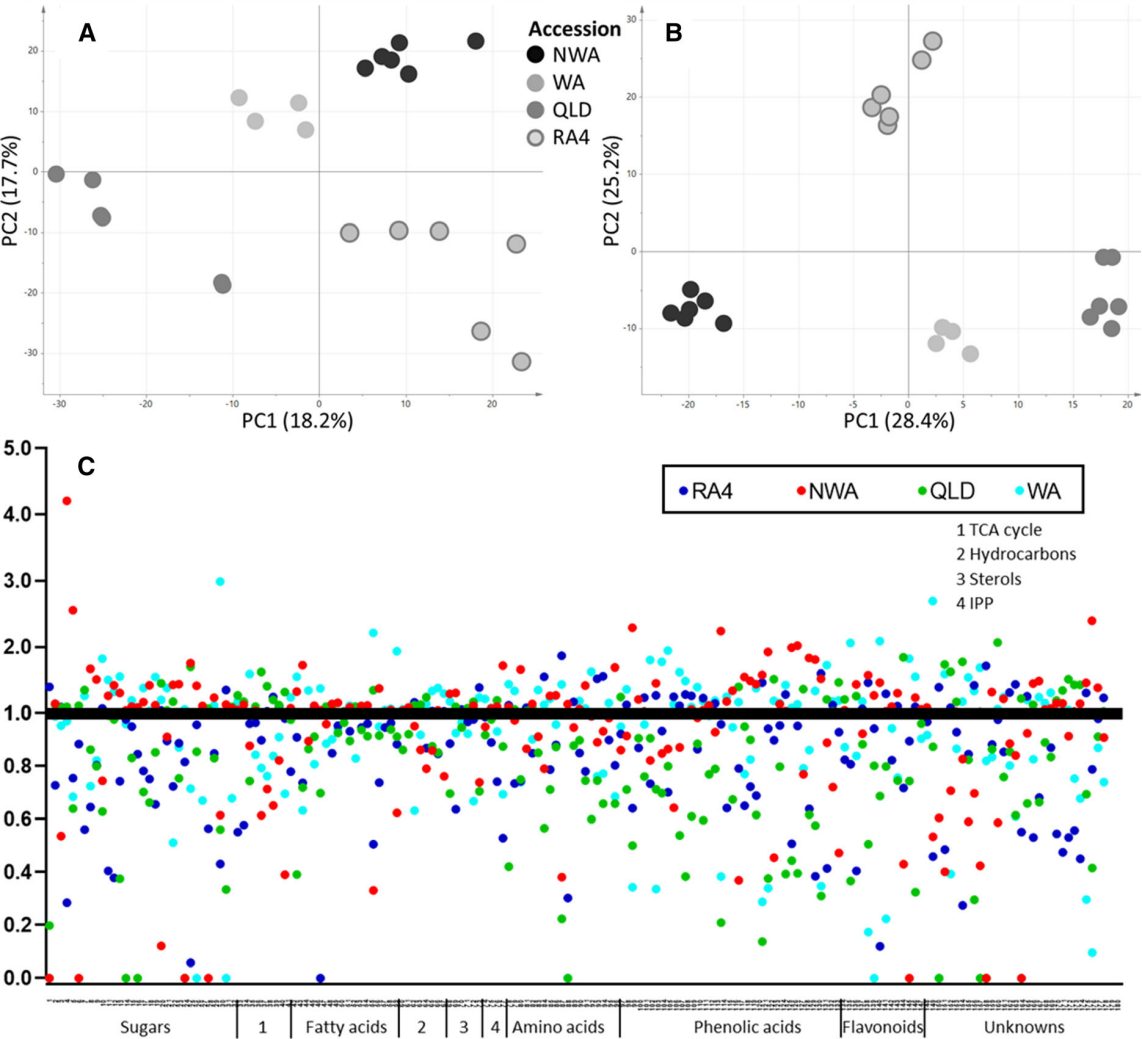
*N. benthamiana* metabolome. PCA score plots were based on (**a**) all molecular features detected and **a** only molecular features significantly different between the four accessions. Biological replicates are displayed separately and include three technical replicates each. **a** Average levels of each metabolite of each accession are displayed as ratios to the overall average of all accessions

The LC–MS metabolite profiling of three wild *N. benthamiana* accessions from Queensland (QLD), Northwest Australia (NWA) and West Australia (WA) and the *N. benthamiana* lab isolate RA4 (RA4) detected 1478 molecular features and showed an average biological variation of ~ 34% (Supplementary Table 1). PCA analysis highlighted the biochemical difference between the lab strain RA4 and the three wild accessions (Fig. 1a). This difference was even more prevalent in the PCA analysis of 725 features, which were significantly different between the four accessions (Fig. 1b). However, the two principle components PC1 (x-axis) and PC2 (y-axis) showed a similar percentage in both score plots, which indicates that the metabolite variability between the wild and lab accessions is similar to the variability between the wild accessions. This metabolic variability, when visualised as a heatmap display, showed that each accession had higher levels of a specific group of molecular features and that some of these features were also higher in other accessions, leading to the almost equal variability explained by the first two PCs (Supplementary Fig. 1). The direct comparison of each wild accession to the lab strain showed that less than a third of the molecular features were significantly different. The closest similarity was between WA and RA4 (25% difference) and the least similarity between QLD and RA4 (33% difference).

**Fig. 2:**
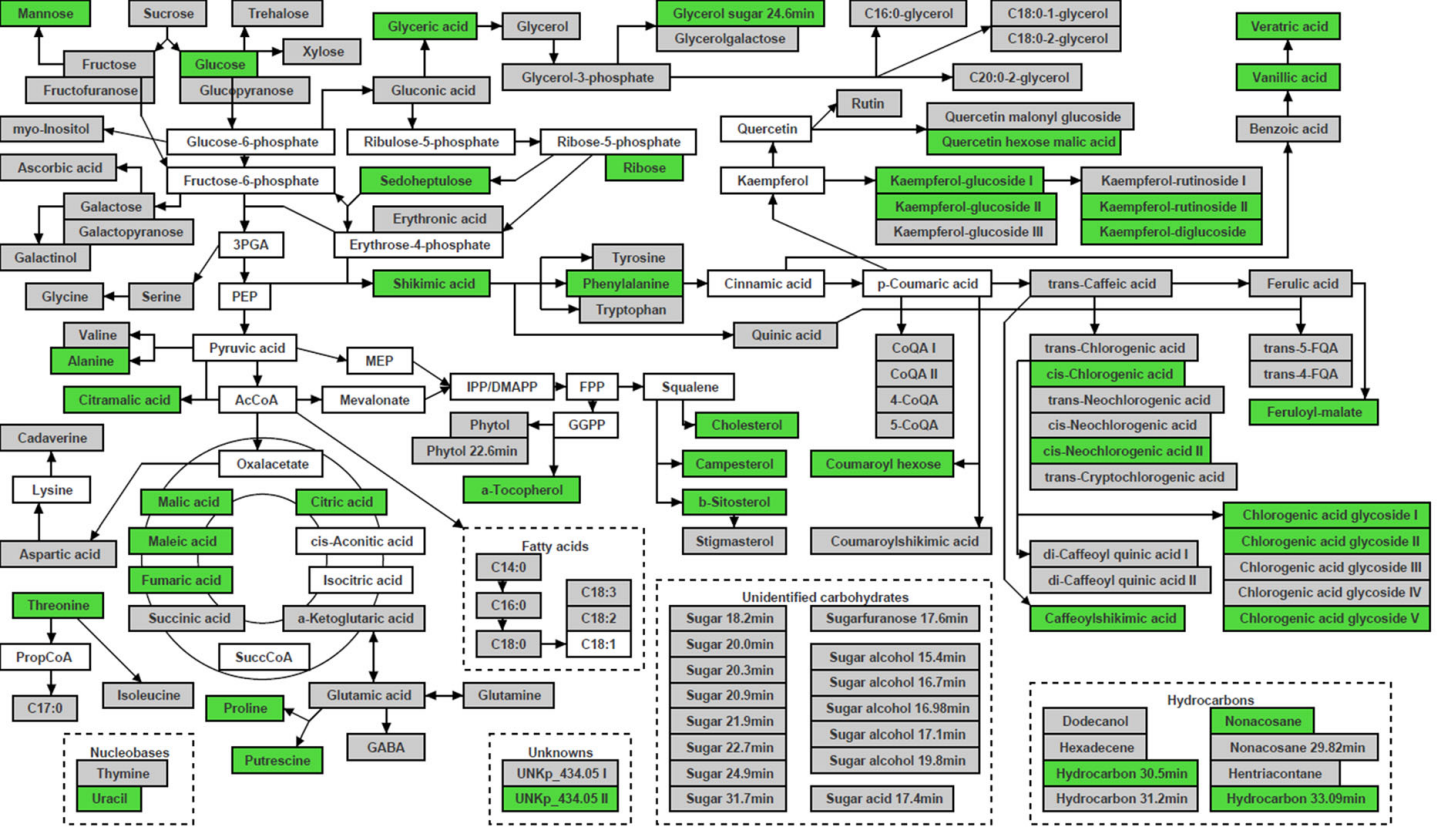
Pathway display of metabolites detected in *N. benthamiana* leaf tissue. Metabolites are colour coded depending on detection (grey), below the limit of detection or not present in the samples (white) and significantly different between any or all four accessions (green)

Metabolite identification showed that the significantly different molecular features represented a wide range of chemical classes such as sugars, TCA cycle intermediates, amino acids, fatty acids and hydrocarbons, phenylpropanoid derived metabolites, phytosterols and isoprenoids (Fig. 2). The levels of these metabolites varied between the accessions. For ease of comparison, the average levels of each accession were compared to the overall average of all four *N. benthamiana* accessions analysed (Fig. 1c). This analysis highlighted that NWA had > 2.5-fold higher levels of galactose, ~ 1.5-fold higher levels of phenolic acids and their quinate esters and the highest levels of shikimic acid (~ 70 µg/g DW) and vanillic acid (~ 5 µg/g DW). WA had > 1.5-fold higher levels of chlorogenic acid glycosides and > twofold higher levels of cell wall related metabolites trehalose, C18:0-glyceride and dodecanol. RA4 showed average levels of phenylpropanoid derived compounds and QLD had the lowest levels of phenolic acids and flavonoids. QLD also showed the lowest levels of amino acids compared to the other accessions.

### Agroinfiltration of *N. benthamiana* accessions

**Fig. 3:**
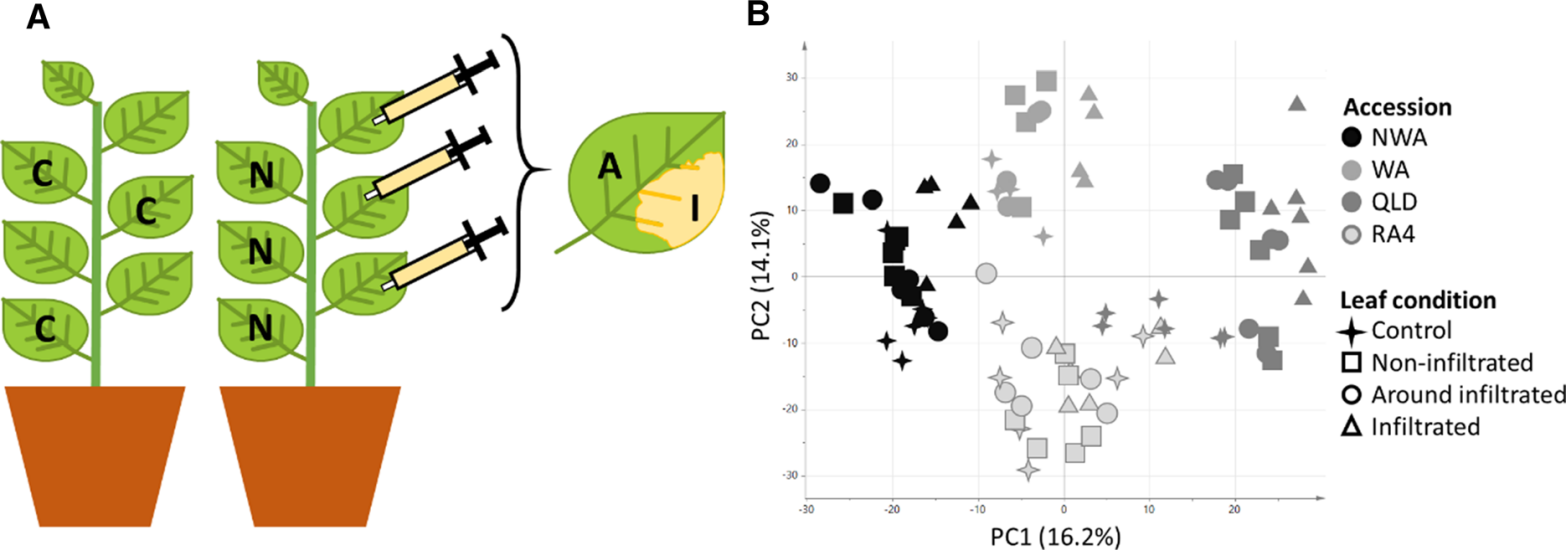
Schematic outline of agroinfiltration of *N. benthamiana* plants (**a**) and PCA analysis of the metabolic effects (**b**). The leaf conditions tested included the infiltrated area (**I**), the area around the infiltrate (**A**), non-infiltrated leaves of the same plant (**N**) and leaves of a non-infiltrated control plant (**C**). Each condition was harvested and analysed as a pool of three leaves per plant. PCA analysis includes data from LC–MS metabolite profiling and shows all biological replicates analysed

To assess the effect of agroinfiltration on the metabolic composition of *N. benthamiana*, the infiltrated leaf area (I) was compared to the leaf around the infiltrated area (A), a non-infiltrated leaf of the same plant (N) and a leaf of a non-infiltrated control plant (C) (Fig. 3a). The comparison included data of all four *N. benthamiana* accessions to assess the generic reaction of *N. benthamiana* plants to the agroinfiltration (Supplementary Table 2). PCA analysis of this data showed that the primary separation of all samples was by accession and then by leaf condition (Fig. 3b). ANOVA indicated that ~ 20% of the molecular features detected were significantly different between the four leaf conditions for all *N. benthamiana* accessions. The dendrogram based on heatmap analysis showed that two thirds of the 298 significant features were significantly higher in the infiltrated leaf area (Supplementary Fig. 2). The other significant molecular features showed a clear pattern specific to the other three leaf conditions. Furthermore, the dendrogram showed that the non-infiltrated areas of the infiltrated plant (A and N) grouped together and were more similar to the control leaves (C) than the infiltrated leaf area (I).

Polar and non-polar extracts of the four leaf conditions were analysed and resulted in the identification of primary (97) and secondary (74) metabolites (Fig. 2, Supplementary Table 3). For more in depth analysis of the metabolites, primary and secondary metabolites were analysed separately. ANOVA results were combined with heatmap displays to show the differences in metabolites levels between the four leaf conditions.

### Changes affecting primary metabolism

**Fig. 4:**
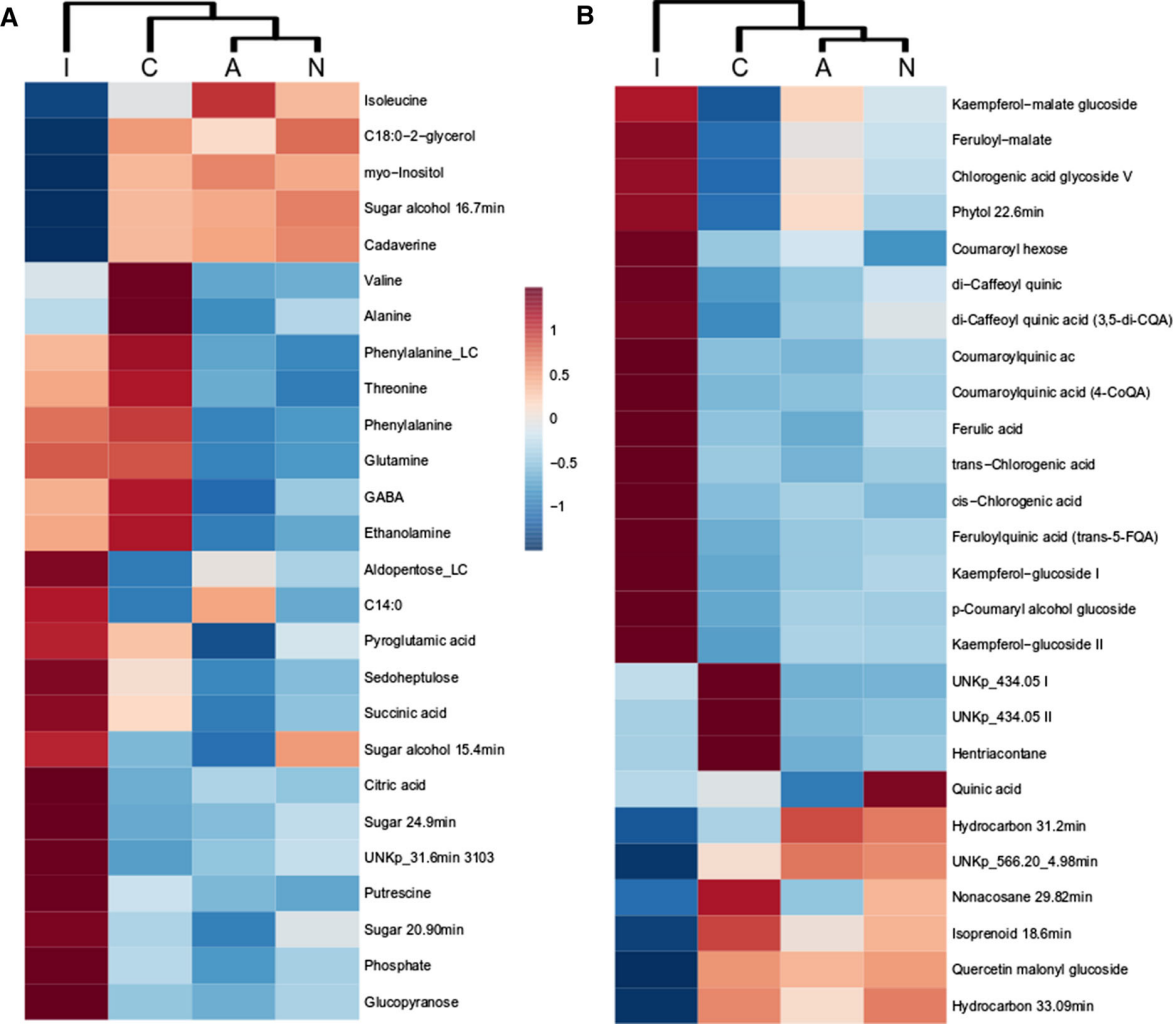
Heatmap of agroinfiltrated and control leaves based on significant primary (a) and secondary (b) metabolites. Data includes four *N. benthamiana* accessions with 4–6 biological replicates per leaf condition. Leaf conditions: non-infiltrated leaf (N), infiltrated leaf area (I), leaf around the infiltrated area (A) and leaf from control plant (C)

Hierarchical analysis of primary metabolites was concurrent with the metabolite profiling and highlighted the highest similarity between leaf area around the infiltrate (A) and non-infiltrated leaves (N), followed by the control leaves (C) and lastly the infiltrated leaf area (I) (Supplementary Fig. 3a). ANOVA determined 26 primary metabolites, which were significantly different between the leaf conditions of all four *N. benthamiana* accessions (Fig. 4a). These metabolites followed three trends including (i) significantly higher, (ii) significantly lower levels in the infiltrated leaf area compared to the other three leaf conditions and iii) similar levels between control and infiltrated leaves. The exception to this were the amino acids valine and alanine, which showed distinct levels in control leaves (up to twofold higher). The metabolites up to 50% significantly lower in the infiltrated leaf area included cadaverine, isoleucine, C18:0–2-glycerol ester, *myo*-inositol and an unknown sugar alcohol. Contrary to this, citric acid, phosphate, putrescine and several unidentified sugars were 1.5- to sixfold significantly higher in the infiltrated leaf area. The metabolites with similar levels between infiltrated and control leaves included amino acids involved in nitrogen fixation, precursor to the phenylpropanoid superpathway as well as sedoheptulose and succinic acid. These metabolites were between 10–70% significantly lower in non-infiltrated leaves and around the infiltrated leaf area.

### Changes affecting secondary metabolites

Secondary metabolites detected in the present study included phytosterols, isoprenoids, hydrocarbons, flavonoids, phenolic acids and their quinic acid esters (Supplementary Fig. 3b). The dendrogram based on 26 significant secondary metabolites showed the same grouping of leaf conditions as the metabolite profiling and the primary metabolites (Fig. 4b). The infiltrated leaf area had significantly higher levels (1.2- to sevenfold) of flavonoids, phenolics and their quinic esters. The leaf around the infiltrated area had ~ 1.4-fold significantly higher levels of kaempferol-malate glucoside, feruloyl-malate, chlorogenic acid glucoside V and phytol. The non-infiltrated leaves had 1.5- to 2.1- fold significantly higher levels of quinic acid compared to the other three leaf conditions. Hentriacontane and two unknowns with the mass 434.05 were 50% significantly lower in control leaves compared to the infiltrated plant. Half of the detected hydrocarbons, an unidentified isoprenoid and quercetin malonyl glucoside were significantly lower (10–40%) in the infiltrated leaf area.

### Metabolic response to agroinfiltration specific to *N. benthamiana* accessions

**Fig. 5:**
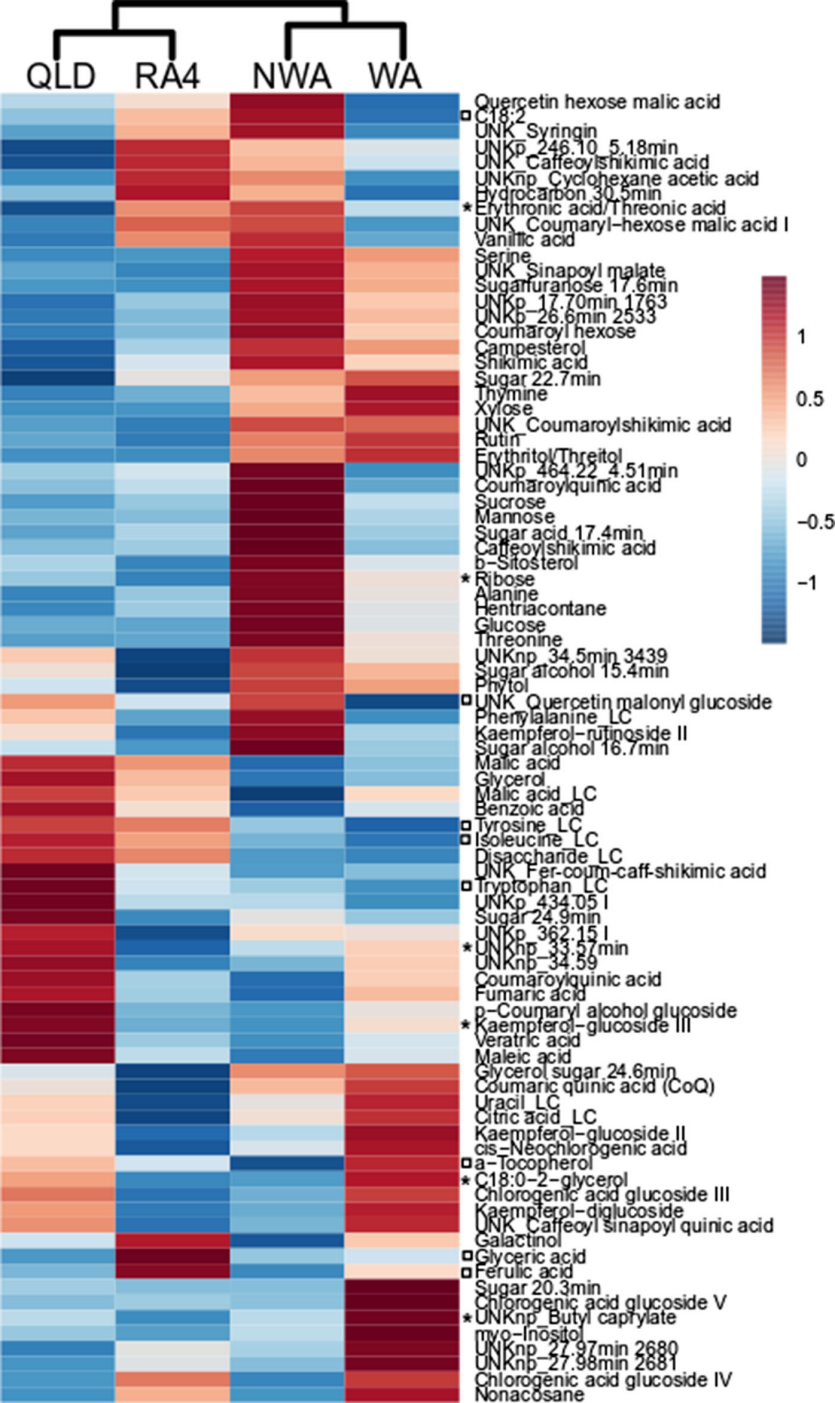
Heatmap of metabolites detected in the infiltrated leaf area. Only metabolites which were significantly different between *N. benthamiana* accessions are displayed. A two-way ANOVA comparison to the control plant highlighted metabolites which showed the same ratio across all accessions (box) and different ratios between accessions (asterix)

The metabolite composition of the infiltrated leaf area was compared between accession as i) a direct comparison of metabolite levels and ii) a ratio to the respective control plant (Fig. 5). The focus of the data analysis was to establish metabolite changes which provide RA4 with improved properties for agroinfiltration compared to the wild accessions. Overall, all four accessions showed ~ 8% of metabolites with the same increase/decrease compared to the control leaf and ~ 7% of metabolites showed different ratios between the accessions. The metabolites with similar trends included ferulic acid and its quinic acid derivative, quinic acid, glyceric acid, glycerol-phosphate and C18:2, and the amino acids tryptophan, tyrosine, glycine and isoleucine. Of these metabolites, ferulic and glyceric acid were observed with the highest levels in the infiltrated leaf area of RA4 (Fig. 5). The direct comparison of the infiltrated leaf area highlighted most other phenylpropanoid derived compounds were significantly lower in RA4.

The metabolites which showed different ratios to the respective control leaf included precursors to phospholipids ethanolamine, C14:0, C18:0–2-glycerol and hexadecane, erythronic acid/threonic acid, ribose and trehalose and the phenylpropanoids kaempferol-glucoside III and di-caffeoyl quinic acid. These metabolites were lower in infiltrated leaf of RA4 with the exception of erythronic acid/threonic acid (Fig. 5).

## Discussion

*N. benthamiana* lab strains possess several features such as viral hypersusceptibility, high transgene expression and ease of cultivation, which makes these plants a prominent and reliable candidate for transient expression of proteins, antibodies and high value metabolites (Bally et al. 2018). However, indigenous metabolic processes, activated by the presence of *Agrobacterium* and the infiltration process*,* can interfere with the synthesis, functionality and extraction of the product of interest. It is therefore crucial to elucidate the metabolic response of *N. benthamiana* to *Agrobacterium* infiltration and understand the chemotypic superiority of *N. benthamiana* lab strains compared to its wild accessions.

The most distinct observation of the present metabolite data was the lack of catechins and other metabolites involved in tannin synthesis. These compounds might be below the limit of detection or not be produced at all in *N. benthamiana* species. Thus, presenting a chemotype more amenable to agro-transformation, as previously detected in sorghum plants with low tannin content (Fresquet-Corrales et al. 2017; Hammerbacher et al. 2018; Kuriyama et al. 2019).

### Species wide response to agroinfiltration

Leaves of all *N. benthamiana* plants were harvested four days post agroinfiltration to determine the metabolite changes occurring at the peak of transient expression, whilst ensuring a healthy leaf condition with minimal chlorotic or necrotic areas (Krenek et al. 2015). The consistent biological variation of all leaf conditions confirmed that the agroinfiltration was performed in a standardised manner among the biological replicates. The metabolite profiling furthermore highlighted that the metabolic response to *Agrobacterium* was localised in the infiltrated leaf area. This was confirmed by the high similarity of the leaf area around the infiltrate to non-infiltrated leaves of the same plant.

For the metabolic response to the agroinfiltration, two aspects have to be taken into consideration. The first aspect is the mechanical damage to the leaf which leads to a local and systemic response of the plant (León et al. 2001; Savatin et al. 2014; Lukaszuk et al. 2016; Chen et al. 2018). The local response consists of cellular repair and establishing a physical barrier against pathogen penetration of the damaged and therefore more easily accessible leaf area. The systemic response provides energy and biosynthesis of primary materials to the wounded area. The second aspect is the cellular defence against the *Agrobacterium* infection (Gohlke and Deeken 2014; Subramoni et al. 2014; Fagard et al. 2014). The specific proteomic immune response of *N. benthamiana* to agroinfiltration includes immune signalling, cell wall remodelling, proteolysis, nutrient depletion from agroinfiltrated area and decrease of primary and photosynthetic metabolism (Grosse-Holz et al. 2018). In many cases, the metabolites involved in the two aspects listed for agroinfiltration overlap e.g. phenylpropanoids and sugars (Bhattacharya et al. 2010; Chen et al. 2018).

In the present study, amino acids levels were lower throughout the agroinfiltrated plant and suggest a plant wide response to the agroinfiltration through a more active protein expression, which was expected as part of the proteomic immune response (Grosse-Holz et al. 2018).

No significant changes in major sugars: fructose, glucose and sucrose were observed in the infiltrated leaf area, despite increased levels of citric acid and succinic acid. This suggests a lack of sugar transport away from the agroinfiltrated area to deplete the nutrient pool for agrobacteria and possibly an increase of energy molecules to provide precursors for the more active TCA cycle and biosynthesis of phenylpropanoid derived metabolites (Grosse-Holz et al. 2018; Chen et al. 2018). Other precursors for the TCA cycle could include catabolism products of long-chain hydrocarbons, which were significantly reduced in the agroinfiltrated area (Herman and Zhang 2016). The TCA cycle could also have been replenished by GABA, which showed no change in the infiltrated leaf area and significantly lower levels in the area around the infiltrate. This would suggest an active transport of GABA from the surrounding area into the infection site. Previous studies observed several functions of GABA during bacterial infection including acting as a precursor for the TCA cycle and quorum quenching. The present data would suggest both these functions are occuring (Bhattacharya et al. 2010; Fagard et al. 2014).

Several of the other detected metabolite changes in the infiltrated leaf area could be related to cell wall remodelling and included sugar and fatty acid components of phospholipids, cadaverine and putrescine involved in cell wall cross-linking of phenolics and chlorogenic acids for lignin synthesis (Bhattacharya et al. 2010; Jancewicz et al. 2016). Phenylpropanoids have been previously reported to have several functions during agroinfiltration. These functions include induction of *vir* genes by hydroxy-cinnamic acids and their gallate derivatives, plant defence mechanisms by catechins, tannins and flavonoids and lignification by chlorogenic acids (Kapila et al. 1997; Bhattacharya et al. 2010; Chen et al. 2018). Chlorogenic acid derivatives were the prevalent subgroup detected in the present study, which suggests that the preferred response to agroinfiltration involved a localised response in the agroinfiltrated area by lignification (Lee et al. 2019). Considering the ratio of the damaged area to the overall infiltrated leaf area, the increase of phenylpropanoid derived compounds was probably related to pathogen defence rather than the mechanical wounding. This hypothesis could be elucidated through future studies including vacuum-infiltration, as this process does not involve mechanical wounding of the leaves.

Further changes in the agroinfiltrated area included an increase of phytol and α-tocopherol. The latter was also increased in the leaf area around the infiltrate. This indicates degradation of chlorophyll in the agroinfiltrated area, which is consisted with transcriptomic data reporting a down-regulation of photosynthesis (Grosse-Holz et al. 2018). The present study also indicates that the catabolic, antioxidant products of the latter are transported to the cells surrounding the agroinfiltrated area.

### Chemotypic advantages of the *N. benthamiana* lab strain RA4

The lab strain RA4 is more amenable for transient expression than wild *N. benthamiana* accessions through its viral hypersusceptibility (Bally et al. 2015). This bears the question whether any other cellular regulations in the lab strain differ from the wild accessions? Previous studies in *N. tabacum* highlighted that the combination of *Rdr1* silencing and virus infection caused a cellular remodelling of plastids in mesophyll cells (Rakhshandehroo et al. 2017). However, the present study showed very similar metabolite changes in the agroinfiltrated area of all *N. benthamiana* analysed, mainly involving the phenylpropanoid pathway located in the cytosol. A direct comparison of metabolite levels in the agroinfiltrated area highlighted a significantly lower amount of phenylpropanoid derived compounds and metabolites involved in cell wall remodelling in the lab strain RA4. This could indicate a reduced lignification of the agroinfiltrated area, a metabolic adaptation of the lab strain due to the repeated presence of *Agrobacterium*. Therefore, the genetic transfer from wild *N. benthamiana* accessions for high value small molecule production could introduce disadvantageous metabolic reprogramming to the lab strain.

### Potential targets for an enhanced plant chassis

The results from the metabolite data indicate potential targets to improve *N. benthamiana* as a chassis for molecular pharming. The most obvious target is the reduction of already low levels of phenylpropanoid derived compounds. A transgenic *N. benthamiana* line with repressed defence-induced lignification could (i) increase the insertion area of Ti vectors and (ii) improve the extraction/purification of the product of interest, which is the most expensive part of transient expression (Nandi et al. 2016; Lee et al. 2019). A complete knock-out of lignification could cause major impairment of plant growth and it is therefore necessary to precisely target effector genes (Xie et al. 2018). Another target could be a thinner, less complex cell wall to facilitate the infiltration process and bioprocessing after the production cycle. As described for the phenylpropanoid derived compounds, the metabolite changes related to the cell wall were induced upon agroinfiltration (Bhattacharya et al. 2010; Jancewicz et al. 2016). Hence, the knock-out of genes related to cell wall cross-linking with cadaverine and putrescine might provide a valuable target.

During the molecular pharming of pharmaceutical protein products, such as vaccines candidates, using *N. benthamiana* leaves, considerable biomass is generated as a waste product. The use of a *N. benthamiana* strain producing high value small molecules as a chassis could combine otherwise separated production lines and add value to the remaining biomass. A potential candidate molecule could be solanesol, an aliphatic terpene alcohol commonly found in solanaceous plants (Gutbrod et al. 2019; Yan et al. 2019). The precursors for this metabolites could be increased by the chlorophyll degradation observed in the present study. Another high value product naturally produced during this degradation process was α-tocopherol (Sathish et al. 2018). Further potential candidate molecules could be lipids and hydrocarbons for biofuels and industrial use. As described for solanesol, the precursors of the TCA cycle that are used for lipids and hydrocarbons are increased during agroinfiltration. This means that intermediary metabolism is primed and only amenable diversion of the precursors into the targeted pathways is needed instead of the large scale engineering of a new pathway (Tan and Lee 2016). In the future, it would also be of interest to carry out the present studies on material that had been subjected the Agro-infection, whereby the transient expression occurs throughout the plant material in a homogenous manner and is not limited predominantly to the localised areas in the vicinity of the infiltration site (Majer et al. 2017).

In conclusion, the present study identified the metabolite changes that arise within *N. benthamiana* leaf material upon agroinfiltration. The comparison of wild and lab accessions elucidated the metabolic differences that exist between lines and highlights the utility of the present lab accession for transient expression using *Agrobacterium.* These metabolite changes in the RA4 lab strain included a lack of tannins, lower phenylpropanoid levels and increased TCA cycle intermediates. The data presented could be used to direct further improvements to our present *N. benthamiana* chassis being used for transient expression of valuable products. It may also facilitate improved down-stream processing approaches and add value through the processing of the remaining biomass.

## Supplementary Information

Below is the link to the electronic supplementary material.

Supplementary file1 (PDF 485 kb)

Supplementary file2 (XLSX 2585 kb)

## Data Availability

All processed data sets are available as appendices. Unprocessed data can be accessed at https://doi.org/10.17632/thb8bsm4kp.1.

## Abbreviations

ANOVA: Analysis of variance
GC–MS: Gas chromatography mass spectrometry
LC–MS: Liquid chromatography mass spectrometry
PC: Principle component
PCA: Principle component analysis
RSD: Relative standard deviation
TCA: Tricarboxylic acid
