# Supplementary material for: Metabolic effects of agro-infiltration on N. benthamiana accessions
Source: Transgenic Res. 2021 Apr 28;30(3):303–15. doi: 10.1007/s11248-021-00256-9 (PMC8080481; doi:10.1007/s11248-021-00256-9)
Supplement: Supplementary file 1 — Supplementary file1 (PDF 485 kb) [file 11248_2021_256_MOESM1_ESM.pdf]

Metabolic effects of agro-infiltration on *N. benthamiana* accessions

Margit Drapal, Eugenia M. A. Enfissi and Paul D. Fraser*

Department of Biological Sciences, Royal Holloway University of London, Egham, United Kingdom

*Corresponding author: P.Fraser@rhul.ac.uk, Tel: +44 1784 443555

Supplementary Figure 1. Heatmaps of untargeted (A) and identified (B) metabolite data of control plants of *N. benthamiana* NWA, WA, QLD and RA4.

Supplementary Figure 2. Heatmap of molecular features significantly different between leaf conditions.

Supplementary Figure 3. Heatmaps of primary (A) and secondary (B) metabolites in agroinfiltrated and control leaves.

Supplementary Table 1. LC-MS metabolite profiling – untargeted data of control plants of *N. benthamiana* NWA, WA, QLD and RA4.

Supplementary Table 2. LC-MS metabolite profiling – untargeted data of agroinfiltrated plants of *N. benthamiana* NWA, WA, QLD and RA4.

Supplementary Table 3. Levels of identified metabolites in agroinfiltrated and control leaves.

Supplementary Table 4. Metabolite library of *N. benthamiana* accessions.

Supplementary Figure 1.

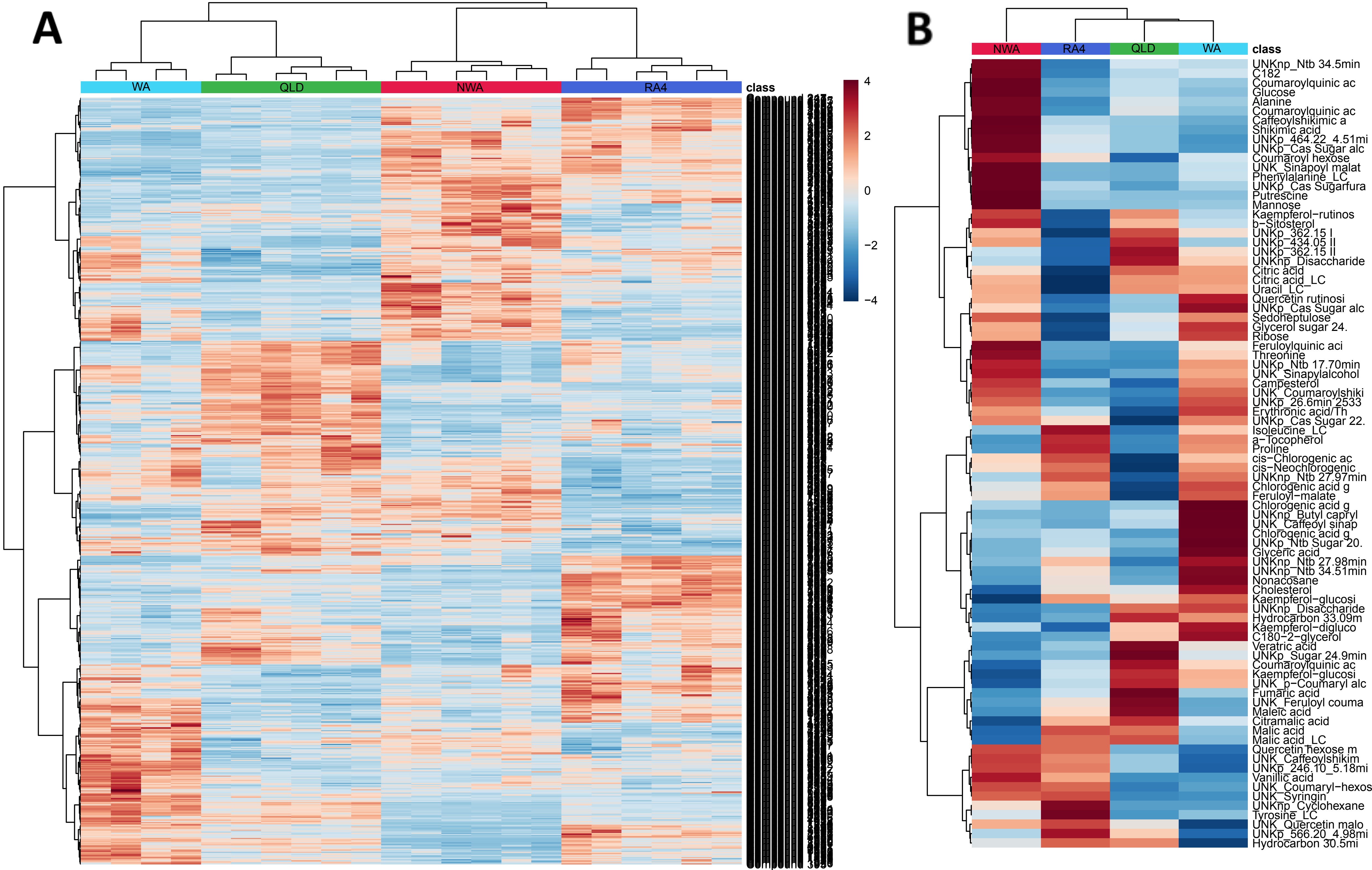

Supplementary Figure 2.

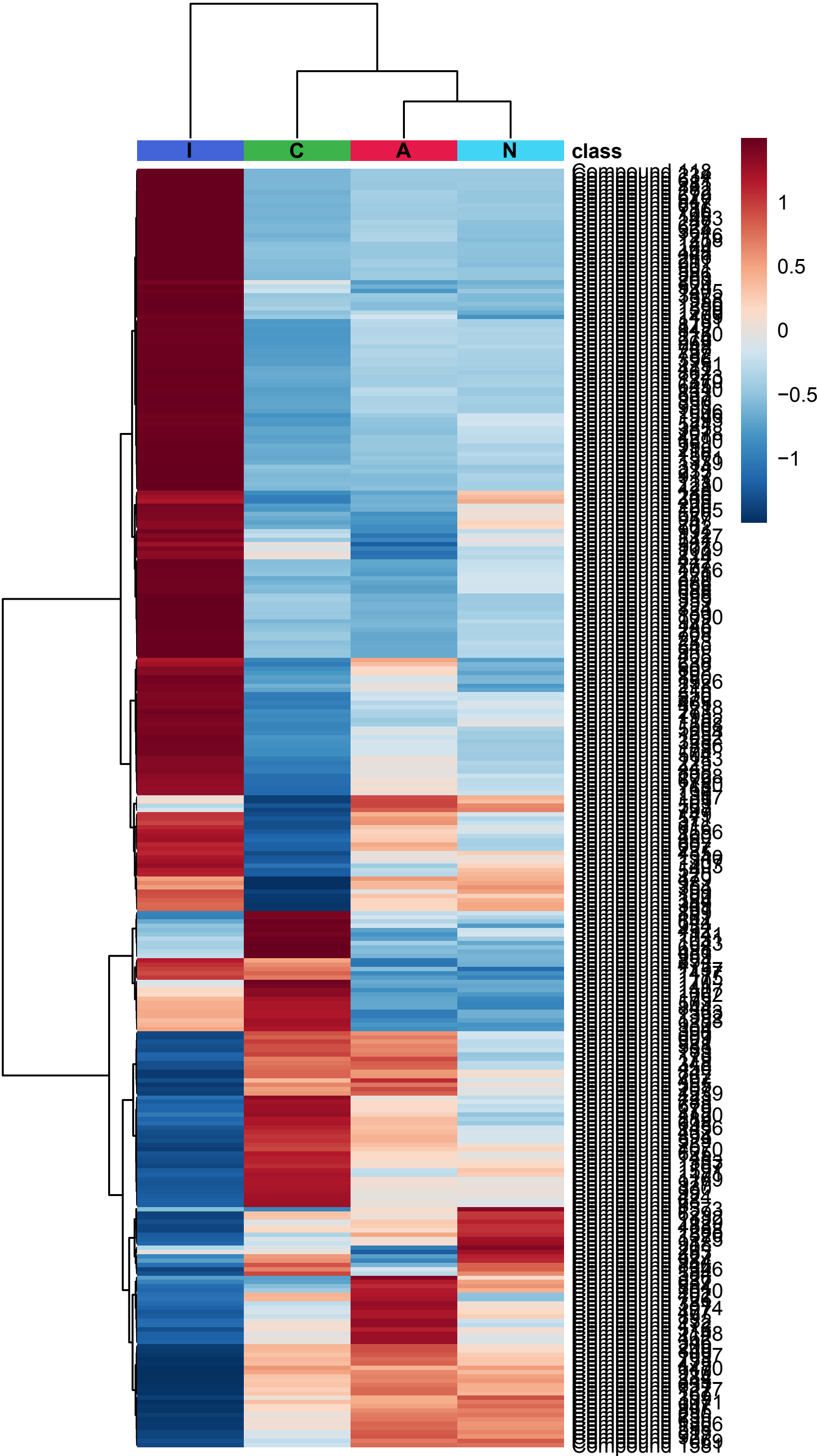

Supplementary Figure 3.

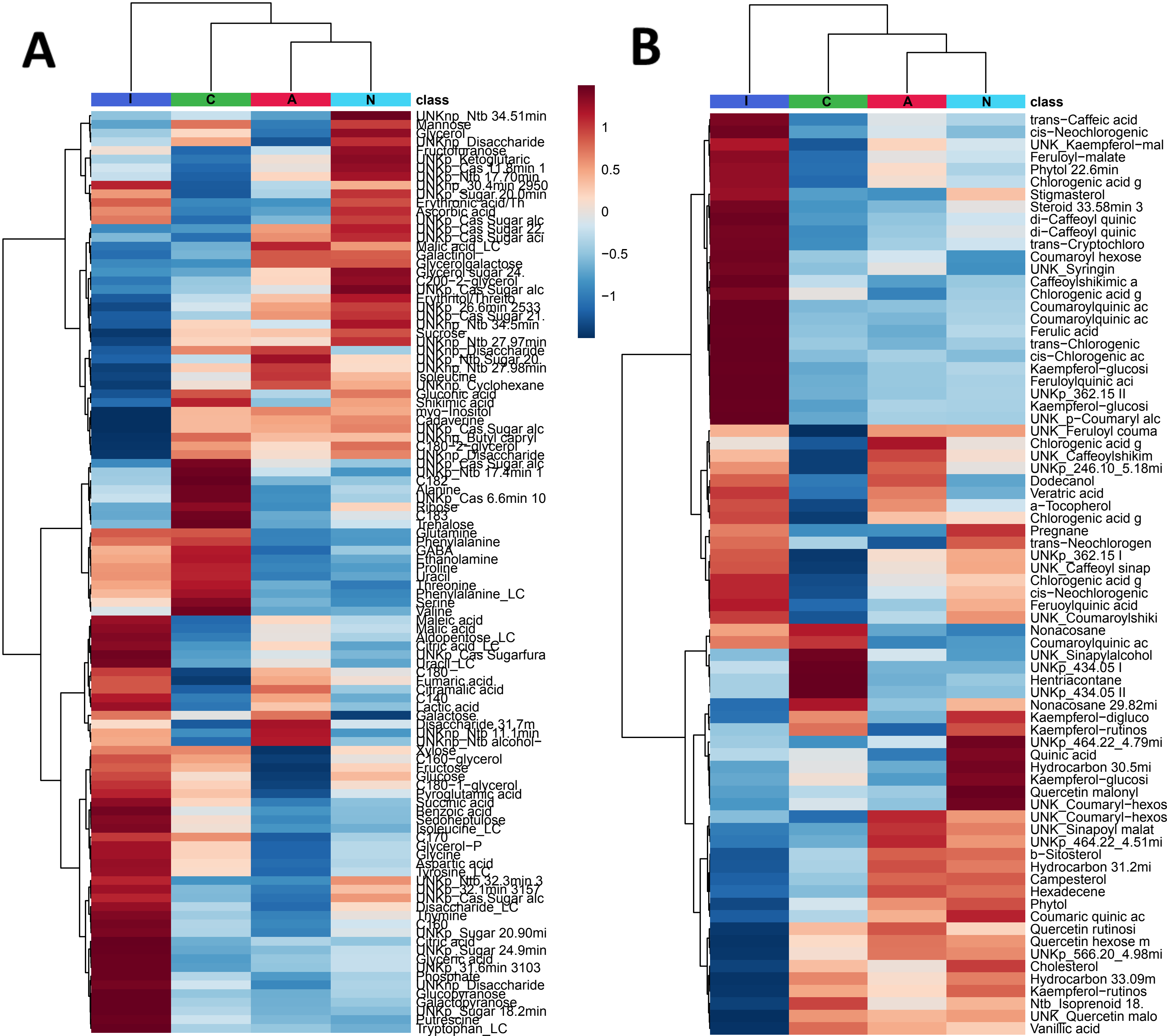

Supplementary Table 4.

[a]	Metabolites	Analytical platform	Identification features (retention time (RT) in min.) RT (GC/MS)	RI (GC/MS)	RT (LC/MS)	m/z [M-H]- (LC/MS)	Identification Extract	Level[c]	Standard[d]	Compound class
1	Fructofuranose	GCMS	18.13	1794.6			Polar	3	No	Sugar
2	Fructose	GCMS	18.53	1824.3			Polar	1	Yes	Sugar
3	Galactinol	GCMS	30.55	2972.4			Polar	1	Yes	Sugar alcohol
4	Galactopyranose	GCMS	19.32	1884.5			Polar	1	Yes	Sugar
5	Galactose	GCMS	19.22	1877.0			Polar	1	Yes	Sugar
6	Gluconic acid	GCMS	20.65	1990.9			Polar	1	Yes	Sugar acid
7	Glucopyranose	GCMS	20.41	1972.0			Polar	3	No	Sugar
8	Glucose	GCMS	19.66	1911.1			Polar	1	Yes	Sugar
9	Mannose	GCMS	18.73	1839.5			Polar	1	Yes	Sugar
10	myo-Inositol	GCMS	21.74	2082.3			Polar	1	Yes	Sugar
11	Ribose	GCMS	16.38	1667.9			Polar	1	Yes	Sugar
12	Sedoheptulose	GCMS	22.11	2118.7			Polar	1	Yes	Sugar
13	Xylose	GCMS	16.06	1646.3			Polar	1	Yes	Sugar
14	LC_Aldopentose	LCMS			0.74	194.06	Polar	3	No	Sugar
15	UNKp_Sugar 21.9min	GCMS	21.93	2098.1			Polar	3	No	Sugar
16	UNKp_Sugar 22.7min	GCMS	22.71	2166.5			Polar	3	No	Sugar
17	UNKp_Sugar acid 17.4min	GCMS	17.44	1744.3			Polar	3	No	Sugar acid
18	UNKp_Sugar alcohol 15.4min	GCMS	15.44	1603.0			Polar	3	No	Sugar alcohol
19	UNKp_Sugar alcohol 16.7min	GCMS	16.68	1688.8			Polar	3	No	Sugar alcohol
20	UNKp_Sugar alcohol 16.98min	GCMS	16.99	1711.1			Polar	3	No	Sugar alcohol
21	UNKp_Sugar alcohol 17.1min	GCMS	17.06	1716.5			Polar	3	No	Sugar
22	UNKp_Sugar alcohol 19.8min	GCMS	19.79	1921.9			Polar	3	No	Sugar alcohol
23	UNKp_Sugarfuranose 17.6min	GCMS	17.63	1758.0			Polar	3	No	Sugar
24	UNKp_Sugar 20.3min	GCMS	20.26	1959.5			Polar	3	No	Sugar
25	UNKp_Sugar 18.2min	GCMS	18.05	1785.4			Polar	3	No	Sugar
26	UNKp_Sugar 20.0min	GCMS	19.97	1935.3			Polar	3	No	Sugar
27	UNKp_Sugar 20.9min	GCMS	20.84	2005.8			Polar	3	No	Sugar
28	UNKp_Sugar 24.9min	GCMS	24.87	2365.1			Polar	3	No	Sugar
29	Sucrose	GCMS	26.87	2563.7			Polar	1	Yes	Sugar
30	Trehalose	GCMS	28.47	2734.9			Polar	1	Yes	Sugar
31	Disaccharide 31.7min	GCMS	31.70	3107.0			Polar	3	No	Sugar
32	LC_Disaccharide	LCMS			0.69	341.12	Polar	3	No	Sugar
33	Citric acid	LCMS/GCMS	18.32	1808.7	0.86	191.03	Polar	1	Yes	TCA cycle
35	Ketoglutaric acid	GCMS	18.54	1827.2			Polar	1	Yes	TCA cycle
36	Succinic acid	GCMS	10.69	1301.4			Polar	1	Yes	TCA cycle
37	Fumaric acid	GCMS	11.39	1345.3			Polar	1	Yes	TCA cycle
38	Maleic acid	GCMS	10.62	1299.1			Polar	1	Yes	TCA cycle
39	Malic acid	LCMS/GCMS	13.62	1482.8	0.72	133.02	Polar	1	Yes	TCA cycle
41	Citramalic acid	GCMS	13.19	1456.1			Polar	1	Yes	TCA cycle
42	Erythritol/Threitol	GCMS	13.89	1500.1			Polar	2	No	Cell wall
43	Erythronic acid/Threonic acid	GCMS	14.21	1521.4			Polar	1	Yes	Sugar acid
44	Ethanolamine	GCMS	10.03	1264.5			Polar	1	Yes	Other
45	Glyceric acid	GCMS	11.05	1324.9			Polar	1	Yes	Sugar acid
46	Glycerol	GCMS	10.10	1269.0			Polar	1	Yes	Cell wall
47	Glycerol sugar 24.6min	GCMS	24.60	2343.2			Polar	2	No	Glyceride
48	Galactosylglycerol	GCMS	24.18	2299.1			Polar	1	Yes	Glyceride
49	Glycerol-3-phosphate	GCMS	16.97	1709.5			Polar	1	Yes	Glyceride
50	C14:0	GCMS	18.62	1832.0			Non-polar	1	Yes	Fatty acid
51	C16:0	GCMS	21.13	2034.1			Non-polar	1	Yes	Fatty acid
52	C16:0-glycerol	GCMS	26.90	2571.3			Non-polar	1	Yes	Fatty acid
53	C17:0	GCMS	21.93	2099.2			Non-polar	1	Yes	Fatty acid
54	C18:0	GCMS	23.38	2225.9			Non-polar	1	Yes	Fatty acid
55	C18:0-1-glycerol	GCMS	28.67	2769.8			Non-polar	1	Yes	Fatty acid
56	C18:0-2-glycerol	GCMS	28.66	2755.9			Non-polar	1	Yes	Fatty acid
57	C18:2	GCMS	23.02	2193.0			Non-polar	1	Yes	Fatty acid
58	C18:3	GCMS	23.15	2203.2			Non-polar	1	Yes	Fatty acid
59	C20:0-2-glycerol	GCMS	30.38	2952.5			Non-polar	1	Yes	Fatty acid
60	Dodecanol	GCMS	14.70	1553.9			Non-polar	1	Yes	Hydrocarbon
61	Hentriacontane	GCMS	31.50	3086.1			Non-polar	1	Yes	Hydrocarbon
62	Hexadecene	GCMS	18.59	1829.2			Non-polar	1	Yes	Hydrocarbon
63	Hydrocarbon 30.5min	GCMS	30.50	2960.4			Non-polar	3	No	Hydrocarbon
64	Hydrocarbon 31.2min	GCMS	31.20	3049.5			Non-polar	3	No	Hydrocarbon
65	Hydrocarbon 33.09min	GCMS	33.09	3272.8			Non-polar	3	No	Hydrocarbon
66	Nonacosane	GCMS	29.48	2849.0			Non-polar	1	Yes	Hydrocarbon
67	Nonacosane 29.82min	GCMS	29.82	2885.5			Non-polar	2	No	Hydrocarbon
68	Cholesterol	GCMS	31.92	3135.6			Non-polar	1	Yes	Sterol
69	Campesterol	GCMS	32.77	3236.1			Non-polar	1	Yes	Sterol
70	b-Sitosterol	GCMS	33.45	3317.7			Non-polar	1	Yes	Sterol
71	Stigmasterol	GCMS	32.97	3260.2			Non-polar	1	Yes	Sterol
72	UNKnp_Pregnane	GCMS	18.73	1839.7			Non-polar	3	No	Sterol
73	Steroid 33.58min 3333	GCMS	33.58	3333.0			Non-polar	3	No	Sterol
74	a-Tocopherol	GCMS	31.79	3120.5			Non-polar	1	Yes	Isoprenoid
75	Ntb_Isoprenoid 18.6min	GCMS	18.86	1865.6			Non-polar	3	No	Isoprenoid
76	Phytol	GCMS	18.53	1824.1			Non-polar	1	Yes	Isoprenoid
77	Phytol 22.6min	GCMS	22.58	2155.7			Non-polar	2	No	Isoprenoid
78	Alanine	GCMS	7.40	1109.5			Polar	1	Yes	Amino acid
79	Aspartic acid	GCMS	14.09	1513.1			Polar	1	Yes	Amino acid
80	Cadaverine	GCMS	8.10	1148.5			Polar	1	Yes	Amino acid
81	GABA	GCMS	14.24	1523.4			Polar	1	Yes	Amino acid
82	Glutamine	GCMS	17.73	1764.9			Polar	1	Yes	Amino acid
83	Glycine	GCMS	10.69	1302.8			Polar	1	Yes	Amino acid
84	Isoleucine	LCMS/GCMS	10.46	1289.4	0.96	130.09	Polar	1	Yes	Amino acid
86	Phenylalanine	LCMS/GCMS	15.52	1608.7	2.38	164.08	Polar	1	Yes	Amino acid
88	Proline	GCMS	10.55	1294.4			Polar	1	Yes	Amino acid
89	Putrescine	GCMS	17.25	1730.0			Polar	1	Yes	Amino acid
90	Pyroglutamic acid [b]	GCMS	14.11	1514.7			Polar	1	Yes	Amino acid
91	Serine	GCMS	11.56	1355.2			Polar	1	Yes	Amino acid
92	Threonine	GCMS	11.96	1379.5			Polar	1	Yes	Amino acid
94	Tryptophan	LCMS			3.66	203.09	Polar	1	Yes	Amino acid
95	Tyrosine	LCMS			1.51	180.08	Polar	1	Yes	Amino acid
96	Valine	GCMS	9.19	1215.0			Polar	1	Yes	Amino acid
97	Shikimic acid	GCMS	18.20	1799.2			Polar	1	Yes	Phenylpropanoid
98	Quinic acid	LCMS			0.92	191.06	Polar	1	Yes	Phenylpropanoid
99	trans-Caffeic acid	GCMS	22.22	2123.4			Polar	1	Yes	Phenylpropanoid
100	Caffeoylshikimic acid	LCMS			4.07	335.08	Polar	2	No	Phenylpropanoid
101	di-Caffeoyl quinic acid	LCMS			3.07	515.15	Polar	2	No	Phenylpropanoid
102	di-Caffeoyl quinic acid (3,5-di-CQA)	LCMS			3.38	515.15	Polar	2	No	Phenylpropanoid
103	UNK_Caffeoyl sinapoyl quinic acid	LCMS			5.62	559.09	Polar	2	No	Phenylpropanoid
104	UNK_Caffeoylshikimic acid	LCMS			5.18	335.07	Polar	2	No	Phenylpropanoid
105	Chlorogenic acid glycoside I	LCMS			5.44	515.13	Polar	2	No	Phenylpropanoid
106	Chlorogenic acid glycoside II	LCMS			5.62	515.13	Polar	2	No	Phenylpropanoid
107	Chlorogenic acid glycoside III	LCMS			5.73	515.13	Polar	2	No	Phenylpropanoid
108	Chlorogenic acid glycoside IV	LCMS			5.85	515.13	Polar	2	No	Phenylpropanoid
109	Chlorogenic acid glycoside V	LCMS			6.01	515.18	Polar	2	No	Phenylpropanoid
110	cis-Chlorogenic acid	LCMS			3.96	353.09	Polar	1	Yes	Phenylpropanoid
111	cis-Neochlorogenic acid	LCMS			2.66	353.09	Polar	1	Yes	Phenylpropanoid
112	cis-Neochlorogenic acid II	LCMS			3.25	353.09	Polar	1	Yes	Phenylpropanoid
113	trans-Chlorogenic acid	LCMS			3.41	353.09	Polar	1	Yes	Phenylpropanoid
114	trans-Cryptochlorogenic acid	LCMS			3.59	353.09	Polar	1	Yes	Phenylpropanoid
115	trans-Neochlorogenic acid	LCMS			2.51	353.09	Polar	1	Yes	Phenylpropanoid
116	Coumaric quinic acid (CoQ)	LCMS			3.24	337.10	Polar	2	No	Phenylpropanoid
117	Coumaroyl hexose	LCMS			3.51	325.10	Polar	2	No	Phenylpropanoid
118	Coumaroylquinic acid	LCMS			4.88	337.10	Polar	2	No	Phenylpropanoid
119	Coumaroylquinic acid (4-CoQA)	LCMS			4.05	337.10	Polar	2	No	Phenylpropanoid
120	Coumaroylquinic acid (5-CoQA)	LCMS			4.56	337.10	Polar	2	No	Phenylpropanoid
121	UNK_Coumaroylshikimic acid	LCMS			3.88	319.15	Polar	2	No	Phenylpropanoid
122	UNK_Coumaryl-hexose malic acid I	LCMS			5.44	441.18	Polar	2	No	Phenylpropanoid
123	UNK_Coumaryl-hexose malic acid II	LCMS			5.66	441.18	Polar	2	No	Phenylpropanoid
124	UNK_p-Coumaryl alcohol 4-O-glucoside	LCMS			4.88	311.12	Polar	2	No	Phenylpropanoid
125	Ferulic Acid	LCMS			6.15	193.06	Polar	1	Yes	Phenylpropanoid
126	Feruloyl-malate	LCMS			6.01	309.14	Polar	1	Yes	Phenylpropanoid
127	Feruloylquinic acid (trans-5-FQA)	LCMS			4.36	367.11	Polar	2	No	Phenylpropanoid
128	Feruoylquinic acid (trans-4-FQA)	LCMS			4.75	367.11	Polar	2	No	Phenylpropanoid
129	UNK_Feruloyl coumaroyl caffeoyl shikimic acid	LCMS			6.03	675.13	Polar	2	No	Phenylpropanoid
130	UNK_Sinapoyl malate	LCMS			5.54	339.13	Polar	2	No	Phenylpropanoid
131	UNK_Sinapylalcohol	LCMS			4.72	207.02	Polar	2	No	Phenylpropanoid
132	UNK_Syringin	LCMS			5.63	371.14	Polar	2	No	Phenylpropanoid
133	Kaempferol-diglucoside	LCMS			3.90	609.15	Polar	2	No	Phenylpropanoid
134	Kaempferol-glucoside I	LCMS			4.55	447.09	Polar	1	Yes	Phenylpropanoid
135	Kaempferol-glucoside II	LCMS			4.88	447.10	Polar	1	Yes	Phenylpropanoid
136	Kaempferol-glucoside III	LCMS			5.59	447.10	Polar	1	Yes	Phenylpropanoid
137	Kaempferol-rutinoside I	LCMS			5.07	563.18	Polar	1	Yes	Phenylpropanoid
138	Kaempferol-rutinoside II	LCMS			5.38	593.16	Polar	1	Yes	Phenylpropanoid
139	UNK_Kaempferol-malic acid glucoside	LCMS			6.02	563.16	Polar	2	No	Phenylpropanoid
140	Quercetin hexose malic acid	LCMS			5.49	579.21	Polar	2	No	Phenylpropanoid
141	Quercetin malonyl glucoside	LCMS			5.38	549.21	Polar	1	Yes	Phenylpropanoid
142	Rutin	LCMS			4.96	609.16	Polar	1	Yes	Phenylpropanoid
143	UNK_Quercetin malonyl glucoside	LCMS			5.56	549.20	Polar	2	No	Phenylpropanoid
144	Benzoic acid	GCMS	9.74	1248.5			Polar	1	Yes	Phenylpropanoid
145	Vanillic acid	LCMS			4.45	167.08	Polar	1	Yes	Phenylpropanoid
146	Veratric acid	LCMS			6.72	181.13	Polar	1	Yes	Phenylpropanoid
147	Ascorbic acid	GCMS	19.99	1937.7			Polar	1	Yes	Vitamin
148	Lactic acid	GCMS	6.75	1071.0			Polar	1	Yes	Cell wall
149	Phosphate	GCMS	8.56	1178.0			Polar	1	Yes	Other
150	UNKnp_30.4min 2950	GCMS	30.40	2950.0			Non-polar	4	No	Unknown
151	UNKnp_Butyl caprylate	GCMS	11.36	1342.9			Non-polar	4	No	Unknown
152	UNKnp_Cyclohexane acetic acid	GCMS	10.65	1300.0			Non-polar	4	No	Unknown
153	UNKnp_33.57min	GCMS	33.57	3330.0			Non-polar	4	No	Unknown
154	UNKnp_Disaccharide/Steroid bound compound 33.62min	GCMS	33.62	3328.8			Non-polar	4	No	Unknown
155	UNKnp_Disaccharide/Steroid bound compound 34.51min	GCMS	34.51	3432.3			Non-polar	4	No	Unknown
156	UNKnp_Disaccharide/Steroid bound compound 34.59min	GCMS	34.59	3431.4			Non-polar	4	No	Unknown
157	UNKnp_11.1min 1326	GCMS	11.10	1326.0			Non-polar	4	No	Unknown
158	UNKnp_27.97min 2680	GCMS	27.97	2680.0			Non-polar	4	No	Unknown
159	UNKnp_27.98min 2681	GCMS	27.98	2681.0			Non-polar	4	No	Unknown
160	UNKnp_34.51min 3444	GCMS	34.51	3444.0			Non-polar	4	No	Unknown
161	UNKnp_34.5min 3439	GCMS	34.50	3439.0			Non-polar	4	No	Unknown
162	UNKnp_alcohol-Si-alcohol	GCMS	33.86	3366.1			Non-polar	4	No	Unknown
163	UNKp_26.6min 2533	GCMS	26.60	2533.0			Polar	4	No	Unknown
164	UNKp_31.6min 3103	GCMS	31.60	3103.0			Polar	4	No	Unknown
165	UNKp_32.1min 3157	GCMS	32.10	3157.0			Polar	4	No	Unknown
166	UNKp_11.8min 1369	GCMS	11.76	1369.0			Polar	4	No	Unknown
167	UNKp_6.6min 1060	GCMS	6.60	1060.0			Polar	4	No	Unknown
168	UNKp_17.4min 1745	GCMS	17.40	1745.0			Polar	4	No	Unknown
169	UNKp_17.70min 1763	GCMS	17.70	1763.0			Polar	4	No	Unknown
170	UNKp_32.3min 3178	GCMS	32.30	3178.0			Polar	4	No	Unknown
93	Thymine	GCMS	11.49	1350.2			Polar	1	Yes	Nucleotide
171	Uracil	LCMS/GCMS	11.17	1332.0	0.86	111.0	Polar	1	Yes	Nucleotide
173	UNKp_362.15 I	LCMS			2.83	361.2	Polar	4	No	Unknown
174	UNKp_362.15 II	LCMS			2.92	361.2	Polar	4	No	Unknown
175	UNKp_434.05 I	LCMS			2.84	433.1	Polar	4	No	Unknown
176	UNKp_434.05 II	LCMS			2.96	433.1	Polar	4	No	Unknown
177	UNKp_246.10_5.18min	LCMS			5.18	245.1	Polar	4	No	Unknown
178	UNKp_464.22_4.51min	LCMS			4.51	463.2	Polar	4	No	Unknown
179	UNKp_464.22_4.79min	LCMS			4.79	463.2	Polar	4	No	Unknown
180	UNKp_566.20_4.98min	LCMS			4.98	565.2	Polar	4	No	Unknown

[a] Compound number in Figure 1C

[b] Derivatisation of glutamic acid produces the corresponding TMS derivatives in addition to TMS-pyroglutamic acid derivatives (Kanani H, Klapa MI (2007). Metabolic Engineering, 9 39-51).

[c] Identification confirmed by spectral and chromatographic properties comparison to authentic commercial standards

[d] Level of metabolite identification as in (Bino et al. (2004) Trends in Plant Science, 9:418–425; Sumner et al. (2007) Metabolomics, 3:211-221) *(1) Identified compounds; (2) Putatively annotated compounds; (3) Putatively characterized compound classes; (4) Unknown compounds*
